# Epstein-Barr Virus encoded LMP1 regulates cyclin D1 promoter activity by nuclear EGFR and STAT3 in CNE1 cells

**DOI:** 10.1186/1756-9966-32-90

**Published:** 2013-11-13

**Authors:** Yang Xu, Ying Shi, Qi Yuan, Xuli Liu, Bin Yan, Ling Chen, Yongguang Tao, Ya Cao

**Affiliations:** 1 Cancer Research Institute, Central South University, Changsha, Hunan 410078, China; 2 Key Laboratory of Carcinogenesis and Cancer Invasion, Ministry of Education, Changsha, Hunan 410078, China; 3 Key Laboratory of Carcinogenesis, Ministry of Health, Changsha, Hunan 410078, China; 4 Department of Gastroenterology, The Second Xiangya Hospital, Central South University, Changsha, Hunan 410011, China; 5 Molecular Imaging Center, Central South University, Changsha, Hunan 410078, China

**Keywords:** EGFR, STAT3, Cyclin D1, Epstein–Barr virus, Latent membrane protein 1, Nasopharyngeal carcinoma

## Abstract

The principal Epstein–Barr virus (EBV) oncoprotein, latent membrane protein 1 (LMP1) is strongly associated with nasopharyngeal carcinoma (NPC), a prevalent cancer in China. The epidermal growth factor receptor (EGFR) is important in carcinogenesis, as it is a ubiquitously expressed receptor tyrosine kinase. Signal transducer and activator of transcription 3 (STAT3) is a master transcriptional regulator in proliferation and apoptosis. Our previous study demonstrated that the nuclear EGFR could bind to the cyclin D1 promoter directly in the presence of LMP1, and the correlation between EGFR and STAT3 in NPC remains to be further explored. Here, we have shown that the interaction of EGFR and STAT3 increased in the nucleus in the presence of LMP1. LMP1 promoted both EGFR and STAT3 binding to the promoter region of cyclin D1, in turn, enhancing the promoter activity of cyclin D1. Furthermore, we demonstrated that both transcriptional activity and mRNA levels of cyclin D1 were decreased by small molecule interference of EGFR and STAT3 activity. These findings may provide a novel linkage between the EGFR and STAT3 signaling pathways and the activation of cyclin D1 by LMP1 in the carcinogenesis of NPC.

## Introduction

Epstein-Barr virus (EBV) is a ubiquitous herpes virus that is linked to multiple malignancies, including Burkitt’s lymphoma, Hodgkin’s disease, gastric cancer esophageal cancer cervical cancer and prostate cancer and nasopharyngeal carcinoma (NPC) [1-9]. Latent membrane protein 1 (LMP1) encoded by EBV functions as an essential factor in EBV-induced cell transformation and is expressed in many of the malignancies associated with EBV. LMP1 protein is detected in approximately 60 percent of tissue samples from patients with NPC [10,11], while LMP1 mRNA is detected in nasopharyngeal swabs in over 90% of NPC patients by RT-PCR [12,13]. The frequent expression of LMP1 in undifferentiated NPC points to a role for this viral oncoprotein as a key molecule in NPC pathogenesis [14-19].

Elevated amounts of the epidermal growth factor receptor (EGFR) at both the protein and mRNA levels are detected in the epithelial cell carcinomas including NPC, and its expression correlates with the levels of LMP1 [20]. Our earlier research reports that LMP1 may increase both expression and phosphorylation levels of EGFR [21,22] and that LMP1 could regulate the nuclear accumulation of EGFR in a dose-dependent manner quantitatively and qualitatively [23]. We also showed that nuclear EGFR could bind to the cyclin D1 promoter directly and transactivate the cyclin D1 promoter by LMP1 in NPC. Many factors such as the epidermal growth factor, the DNA damage factor, ultraviolet irradiation, radiation and cetuximab increase EGFR translocation into the nucleus [24-29]. These findings clearly indicate that EGFR may act as a new factor that directly target genes related to cellular transformation, cell cycle regulation, DNA damage repair and replication.

Signal transducer and activator of transcription 3 (STAT3) is a member of the STAT family of cytoplasmic proteins that is constitutively active in many human cancers [30,31]. Upon stimulation by cytokines or growth factors, STAT3 translocates into the nucleus to upregulate numerous target genes, such as cyclin D1, c-fos, c-Myc, Bcl-XL, and VEGF, stimulating cell proliferation and preventing apoptosis. Overexpression and activation of STAT3 is strongly associated with NPC [32-34]. Our previous finding showed that EBV LMP1 stimulates the phosphorylation of STAT3 at both tyrosine 705 (Tyr 705) and serine 727 (Ser 727) [35]. Furthermore, we demonstrated that LMP1 signals through the Janus kinase 3 (JAK3) and extracellular signal-regulated kinase 1/2 (ERK1/2) pathways upon the activation (or transactivation) of STAT3. LMP1 may induce vascular endothelial growth factor (VEGF) expression via the JAK/STAT and mitogen-activated protein kinase (MAPK)/ERK signaling pathways [34]. The relationship between LMP1 regulated STAT3 and other target genes remain unclear.

Cyclin D1 is a key regulatory protein at the G1/S checkpoint of the cell cycle. A recent census concluded that cyclin D1 gene amplification and overexpression are present in breast cancer, lung cancer, melanoma and oral squamous cell carcinomas [30,36,37]. Our previous studies have shown that LMP1 can activate cyclin D1 gene expression [38], upregulate the promoter activity of cyclin D1 by inducing c-Jun/Jun B heterodimers [39] and via EGFR transcriptional activity as well as transcriptional intermediary factor 2 (TIF2) interaction [40] in NPC cell lines. Therefore, we explored whether LMP1 regulated transactivation of the cyclin D1 promoter via activated EGFR and STAT3 in NPC would provide a new link in understanding the mechanisms of carcinogenesis and progression of NPC.

In this study, we found that LMP1 promoted the interaction of EGFR and STAT3 in the nucleus. The nuclear EGFR and STAT3 could target the cyclin D1 promoter directly, in turn, upregulating the cyclin D1 promoter activity and mRNA level. Furthermore, knockdown of EGFR and STAT3 decreased cyclin D1 promoter activity. Our results provide a novel linkage between deregulated EGFR signaling and the activation of cyclin D1 gene expression induced by LMP1 in NPC tumorigenesis.

## Material and methods

### Cell lines

CNE1 is an LMP1-negtive, poorly differentiated NPC cell line. CNE1- LMP1 is a stably transfected cell line, established by introducing LMP1 cDNA into CNE1 cells, and the cell line stably expressing LMP1 [17,34,41-43]. Two cell lines were grown in RPMI 1640 (GIBCO BRL, U.S.A.), containing 10% fetal calf serum and 100 U/ml penicillin/streptomycin, and all cell lines grew, at 37°C under 5% CO_2_ and 95% air at 99% humidity.

### Plasmids

Plasmid (pCCD1-Luc), kindly provided by Dr. Strauss M, contained 3.9 kb of the human cyclin D1 promoter cloned into the multiple cloning sites of pBSK+, driving the gene expression for firefly luciferase. The pcDNA3.1-EGFR expression plasmid was constructed by cloning the whole EGFR coding fragment into *XhoI* sites of the pcDNA3.1 vector. Expression plasmid for dominant negative mutant of EGFR (EGFR-DN) had a deletion of 533 amino acids at the N terminus, which competitively inhibited the activation of EGFR, and was cloned into pcDNA3.1. The pSG5-STAT3 was obtained from whole STAT3 coding fragment cloned into *XhoI* sites of the pSG5 vector. Expression plasmid for dominant negative mutant of STAT3 (STAT3β) had a deletion of 55-residue in C-terminal transactivation domain of STAT3 and replaced by seven unique C-terminal residues (CT7) [44]. The EGFR and STAT3 motif mutation (designated as pD1-mut-Luc) from pCCD1-Luc were generated by PCR based on an overlap extension technique. The primers used for generating mutations were: 5′- CTCCACCTCACCCCCTAAAT-3′ and 5′-AGGGATGGCTTTTGGGCTCT -3′. PCR-amplified fragments carrying the desired mutations were then cloned into *Xba I* sites of the pBSK + vector. The construction of expected TAKARA Biotechnology completed mutations and the sequencing of integrity of the vector. DNAzyme 1 (DZ1) is an LMP1-targeted DNAzyme that binds and cleaves LMP1 RNA in a highly sequence-specific manner [19]. And the control oligonucleotide of DZ1 (TAKARA, China) was designed by inverting the catalytic core sequence. To monitor transfection efficiency, pRL-SV40 (Promega, U.S.A) was used as an internal control.

### Preparation of cell lysates and cell fractions

For whole cell lysates, 10^7^/ml cultured cells were harvested and washed twice with ice-cold phosphate-buffered saline (PBS), and then lysed in the 500 μl lysis buffer [10 mM Tris–HCl, pH 8.0; 1 mM EDTA, 2% sodium dodecyl sulfate (SDS); 5 mM dithiothreitol (DTT); 10 mM phenylmethyl sulfonylfluoride (PMSF); 1 mM Na3VO4; 1 mM NaF; 10% (vol/vol) glycerol; protease inhibitors cocktail tablet (Roche, Switzerland)] for 30 min on ice and centrifuged at 15,000 × g for 10 min. The supernatant was collected and stored at -70°C until used.

For Preparation of cytoplasmic and nuclear fractions, 10^7^/ml cells were washed with PBS and suspended in 200 μl of lysis buffer (10 mM Hepes, pH 7.9; 10 mM KCl; 0.1 mM EDTA; 0.1 mM EGTA; 1 mM DTT; 0.5 mM PMSF; and protease inhibitor cocktail). The cells were incubated on ice for 15 min, after which 6.5 μl of 12.5% NP-40 was added; the contents were mixed and then centrifuged for 1 min at 12,000 rpm. The supernatant was saved as cytoplasmic fraction. The pellet was resuspended in 12.5 μl of ice-cold nuclear extraction buffer (20 mM Hepes, pH 7.9; 0.4 M NaCl; 1 mM EDTA; 1 mM EGTA; 1 mM DTT; 1 mM PMSF; and protease inhibitor cocktail) and incubated on ice for 40 min with mixing every 10 min, then they were centrifuged for 5 min at 12,000 rpm at 4°C. The supernatant was saved as nuclear fraction. The cytosolic and nuclear fractions were stored at -70°C until used.

### Western blot analysis

Fifty microgram (μg) of the total proteins from cell preparations were separated on 10% SDS- polyacrylamide gel electrophoresis and then electrotransfered onto the nitrocellulose membrane. The membranes were blocked with buffer containing 5% non-fat milk in PBS with 0.05% Tween-20 (PBST) for 2 hrs, and incubated with different primary antibodies (anti-EGFR or anti-STAT3) overnight at 4°C. After second wash with PBST, the membranes were incubated with anti-rabbit (sc-2004, Santa Cruz, U.S.A.) or anti-mouse (sc-2005, Santa Cruz, U.S.A.) horseradish peroxidase- conjugated secondary antibody for 1 hr. at room temperature and color was developed with the enhanced chemiluminescence detection kit (ECL, Pierce, U.S.A.), then, and followed by exposure to autoradiographic film. The antibodies used were as follows: EGFR (sc-03-G, Santa Cruz, U.S.A.), p-EGFR (sc-12351, Santa Cruz, U.S.A.), STAT3 (#9132, Cell Signaling Technology, U.S.A.), p-STAT3 (#9131, Cell Signaling Technology, U.S.A.), β-actin (sc-8432, Santa Cruz, U.S.A.), α-tubulin (sc-5286, Santa Cruz, U.S.A.), Nucleolin (sc-8031, Santa Cruz, U.S.A.), cyclin D1 (Cat# 2261–1, Epitomics, U.S.A.).

### Co-immunoprecipitation analysis and immunoblotting analysis

Cell extracts were prepared with harvested cells from CNE1 and CNE1-LMP1 lysed in an immunoprecipitation (IP) lysis buffer (50 mM Tris–HCl, 150 mM NaCl, 10% NP-40, 1 mM EDTA, 10% glycerol, 10 mM NaF, 1 mM Na3VO4, 1 mM DTT, 1 mM PMSF, and protease inhibitor cocktail tablet). Two milligram (mg) of protein prepared were mixed with 40 μl of protein A-Sepharose beads (Sigma, U.S.A.) in the IP assay buffer (1× PBS, 0.5% Nonidet P-40, 0.5% sodium deoxycholate, 0.1% SDS), incubated at 4°C for 2 hrs with gentle agitation and centrifuged for 10 min at 2,000 rpm for preclearing. The recovered supernatant was incubated with either 2 μg of anti-EGFR or 2 μg of anti-STAT3in the presence of 1× protease inhibitors at 4°C overnight with mild shaking. Followed by addition of 50 μl of Protein A-Sepharose beads and the incubation were continued for 2 hrs at 4°C with gentle shaking. Then, Protein A-precipitated protein complex was recovered by centrifugation for 10 sec. at 12,000 rpm and followed washed three times with IP assay buffer, the harvested beads were resuspended in 30 μl of 2× SDS PAGE sample buffer were boiled for 5 min. to release the bound protein. A 20 μg aliquot of cell lysate was used as an input control. The samples were then analyzed by Western blot. Antibodies for Western blot detection were EGFR IgG antibody and STAT3 IgG antibody.

### Transient transfection and luciferase assay

Cells were cultured in 24-well plates at a density of 1 × 10^5^ per well overnight and were transfected with Lipofectamine™ 2,000 (Invitrogen, U.S.A.) as the manufacturer’s instructions. Each transfection contained 800 ng/well of pCCD1-Luc or pD1-mut-Luc firefly luciferase reporter and 80 ng/well of internal control pRL-SV40 or contained 400 ng/well of firefly luciferase reporter and 80 ng/well of internal control pRL-SV40 together with 200 ng/well of each expression plasmid or blank expression plasmid necessary to normalize the amount of DNA transfected. Twenty-four hrs. after transfection, cells were harvested at 36 hrs. after transfection and lysates were analyzed for luciferase activity using the Dual Luciferase Reporter assay (Promega, U.S.A.) according to the manufacturer’s directions with a GloMax™ Microplate Luminometer (Promega, U.S.A.). The luciferase reporter plasmids were co-transfected with pRL-SV40 to correct for variations in transfection efficiency. The relative luciferase activity normalized to the value of pRL-SV40 activity. Results were expressed as fold induction of pCCD1-Luc activity in CNE1 cells, which was assigned a value of 1. WHI-P131, PD98059 and AG1478 inhibited the activities of cyclin D1 induced by stable expression LMP1. CNE1-LMP1 cells were transfected with cyclin D1 promoter-reporter construct and Renilla luciferase plasmid as an internal control. The data represent the mean ± SD of the three independent experiments performed in triplicate.

To observe WHI-P131, PD98059 and AG1478 inhibiting the activities of cyclin D1 induced by stable expression LMP1, 24 hrs. after transfection, cells were treated with WHI-P131 (Calbiochem, U.S.A. ), PD98059 (Cell Signalling Technology, U.S.A. ), AG1478 (Cell Signalling Technolgoy, U.S.A.) or 0.1% DMSO for 2 hr. Cells were harvested at 26 h after transfection and subjected to the luciferase assay. Empty firefly reporter vector served as the negative control.

### Electrophoretic mobility shift assay (EMSA)

EMSA for EGFR/STAT3 binding to cyclin D1 was performed using the LightShift™ Chemiluminesent EMSA kit (Pierce, U.S.A ) and was conducted according to the manufacturer’s protocol. Briefly, Double-stranded oligonucleotides, were labeled using the biotin 3′end labeling (Invitrogen, U.S.A ). Ten μg of nuclear extracts were incubated with 2 μl biotin-labeled probes in binding buffer for 20 min. at room temperature. Additionally, increasing concentrations of 200- fold of excess of a cold competitive oligonucleotide (biotin- unlabeled probe) and NF-κB biotin-unlabeled probe (as a nonspecific competitive probe) were added to confirm specificity of the interaction. The reaction mixture was then loaded onto 10% non- denaturing polyacrylamide gel containing 0.5× Tris borate (TBE) and electro- phoresed in 0.5× TBE at 4°C prior to visualization according to the manufacturer; Followed by transferred to BiodyneR B Nylon membrane, avidin-HRP to probes, and visualized and quantitated with a PhosphorImager (Bio Rad, U.S.A). All the double-stranded probes were synthesized as follows: for the putative binding site of EGFR in the cyclin D1 promoter: 5′-TCGCTGAGAT*TCTTT*GGCCGTCTG-3′ (wild type) and 5′-TCGCTGAGAT*ACTCG*GGCCGTCTG-3′ (mutated type). For the STAT3 binding site in cyclin D1 promoter: 5′-GTGGCG*TTCTTGGAA*ATGCG- CCCA-3′ (wild type) and 5′-GTGGCG*AGCTTGTGA*ATGCGCCCA-3′ (mutated type).

To verify the involvement of EGFR, STAT3, LMP1 in the complex, DZ1, small molecular inhibitors AG1478, WHI-P 131and PD98059, was added to the mixture containing the nuclear extracts and biotin-labeled probes and incubated at room temperature or on ice for an additional 10 min.

### RNA interference

We used EGFR siRNA and STAT3 siRNA to reduce EGFR and STAT3 gene expression. The siRNA sequences for EGFR (sc-29301, Santa Cruz, U.S.A) and STAT3 (sc-29493, Santa Cruz, U.S.A ), and the negative control siRNA (sc-37007, Santa Cruz, U.S.A ) (silencer negative control) were purchased from Santa Cruz. Cells were plated at 30% to 40% confluency, in RPMI 1640 and 10% FCS. The indicated siRNA (100 pmol EGFR siRNA; and/or 100 pmol of STAT3 siRNA) was transfected in six-well plates using 10 μl Lipofect AMINE as recommended (Invitrogen, U.S.A ) for 6 hrs. in serum-free medium. Medium containing serum was added to bring the concentrations of serum to those indicated above.

To study transcriptional activity of endogenous EGFR and STAT3, cells were transiently cotransfected with pCCD1-Luc, and 10 nM of the noncoding control siRNA as a control.

### RT-PCR and quantitative real-time PCR

Cells were transfected with the specified siRNAs and placed in RPMI 1640 with 5% FCS. Forty-eight hours later, they were harvested for RNA isolation using Trizol (Invitrogen, U.S.A). RNA was reverse transcribed with random primers and SuperScript II reverse transcriptase according to Invitrogen’s protocol. The RT Real-Time SYBR/ROX PCR Master Mix was purchased from TAKARA; and PCR analysis was performed on an Applied Biosystems 7500 Real-Time PCR System, according to the instructions of the manufacturer. The RT-PCRs were performed in duplicates for four independent experiments and the results were normalized to the respective expression levels of actin. The primer sequences were for cyclin D1 (forward) 5′-CTCCACCTCACC- CCCTAAAT -3′ and (reverse) 5′-AGAGCCCAAAAGCCATCC-3′ and for actin (forward) 5′-TTCC- AGCCTTCCTTCCTGGG-3′ and (reverse) 5′-TTGCGC- TCAGGAGGAGCAAT-3′. The amplification product of cyclin D1 was 177 bp. The mean ± SD of three independent experiments is shown.

### Flow cytometry

Flow cytometry was used to quantify cells in each phase of the cell cycle. Cells (2 × 10^5^) were plated into 6-well plates and treated with the indicated siRNAs after 24 hrs. Cells were harvested after an additional 72 hrs, washed with PBS and fixed in 70% ethanol overnight at 4°C. To detect the fluorescent intensity of certain proteins, cells were counterstained in the dark with 50 μg/ml phosphatidyl inositol (PI) and 0.1% ribonuclease A (RNase A) in 400 μl of PBS at 25°C for 30 min. Stained cells were assayed and quantified using a FACSort Flow Cytometer (Becton Dickinson, U.S.A).

### Statistical analysis

All statistical calculations were performed with the statistical software program SPSS ver.10.0. Differences between various groups were evaluated by the Student’s t test. The difference was of statistical significance, when *p* <0.05.

## Results

### LMP1 promoted the interaction of EGFR with STAT3 in NPC cells

**Figure 1:**
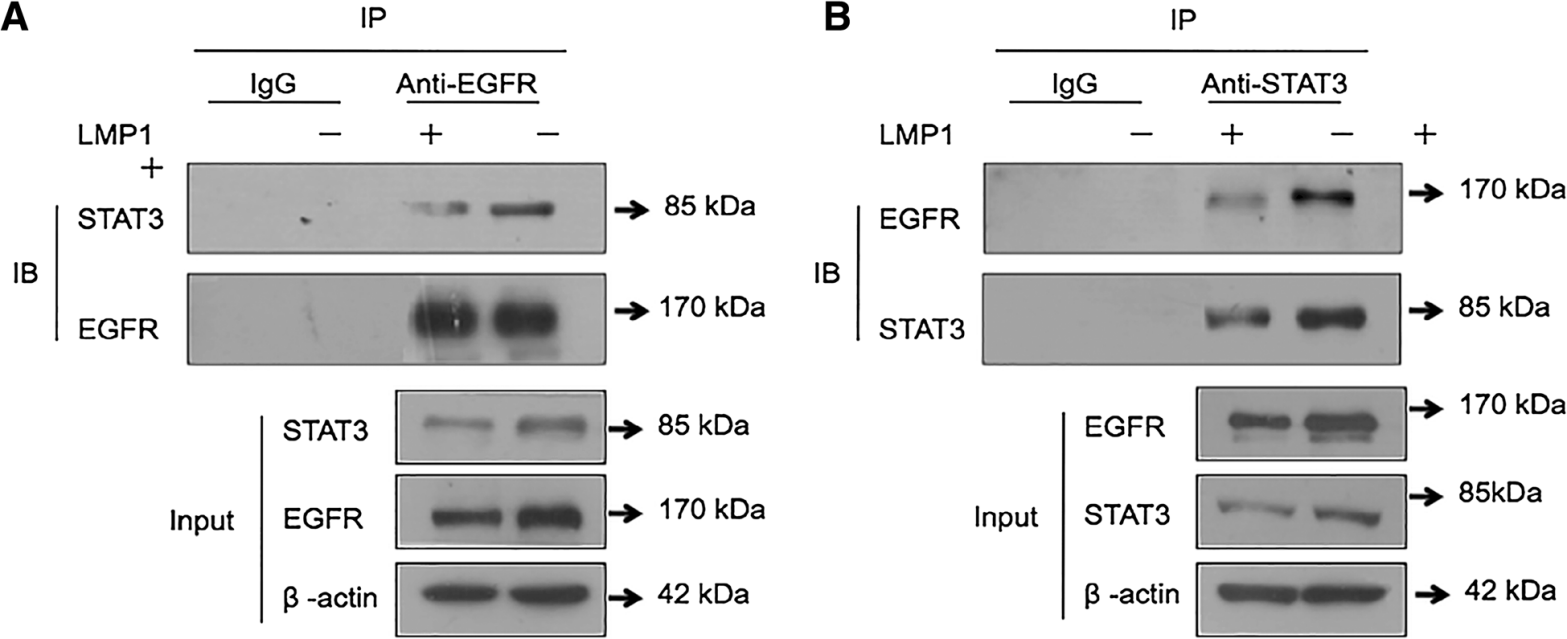
**LMP1 affected the interaction of EGFR and STAT3.** Two mg of protein from cell lysates were immunoprecipitated with an anti-EGFR antibody **(A)** or anti-STAT3 antibody **(B)** and analyzed by Western blotting with a STAT3 and EGFR antibodies. Negative controls included immunoprecipitation with an unrelated antibody (IgG). ®-actin were used as an internal control of Inuput. The bottom panels show the 50 μg of input materials. IP: immunoprecipitation, IB: immunoblot, kDa: kilodalton.

To investigate the possible interaction of EGFR and STAT3 in NPC cells, co-immunoprecipitation (co-IP) with immunoblot analysis was performed. An anti-EGFR antibody pulled down an immunocomplex, and then Western blotting was performed to analyze the STAT3 protein in the complex. Data in Figure 1A show that EGFR interacted with STAT3 using an anti-EGFR antibody while LMP1 increased the interaction of EGFR with STAT3. In addition, Figure 1B indicates that STAT3 interacted with EGFR using an anti-STAT3 antibody, and the interaction of STAT3 with EGFR increased under the regulation of LMP1. Our previous study demonstrated that LMP1 promoted the phosphorylation of STAT3 and EGFR [35,45], Additional file 1: Figure S1 shows that interaction of phosphorylated ETGR with phosphorylated STAT3 increased in the presence of LMP1. These data indicate that EGFR interacts with STAT3 in NPC cells with LMP1 increasing the interaction.

### LMP1 induced EGFR and STAT3 nuclear translocation in NPC cells

**Figure 2:**
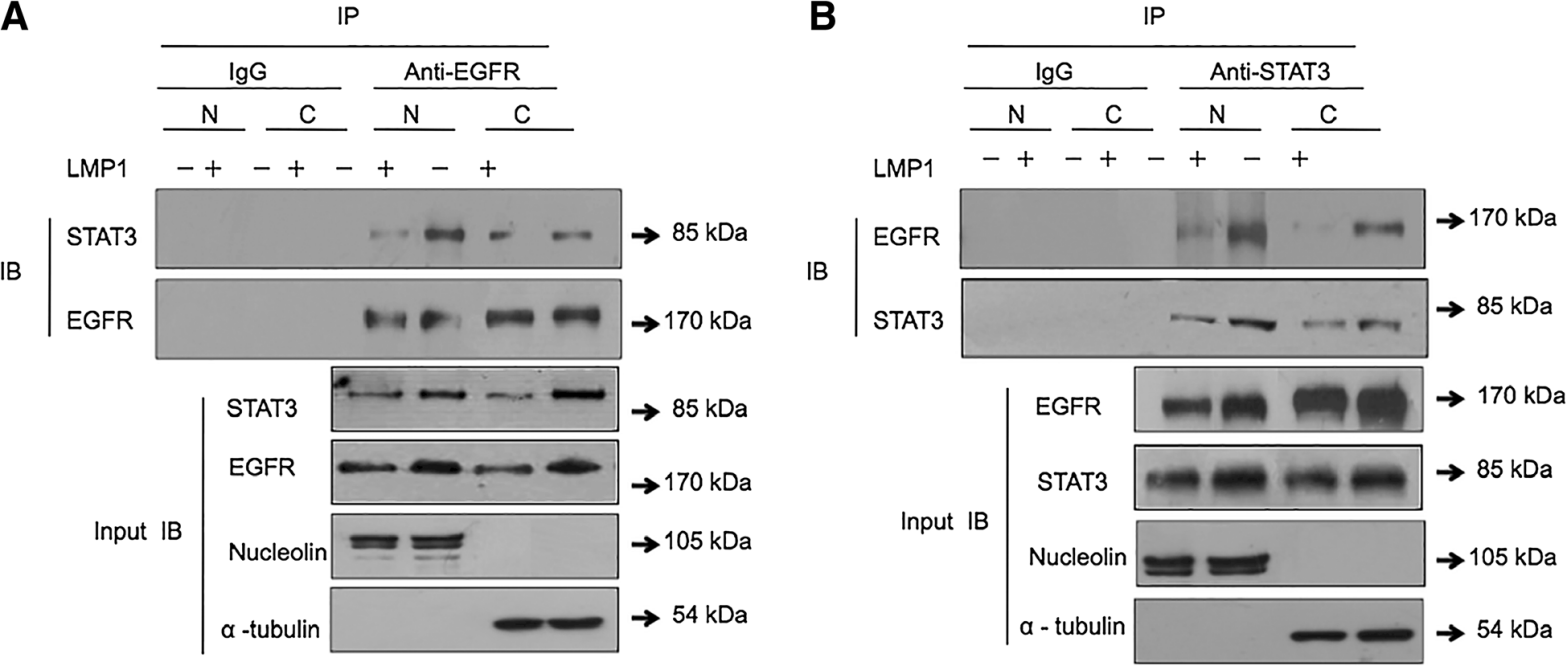
**LMP1 induced co-localization of EGFR and STAT3 in the nucleus.** Endogenous association of EGFR **(A)** with STAT3 **(B)** in NPC cells without or with LMP1 expression. Equal amounts of fractionated cellular proteins were immunoprecipitated with an anti-EGFR or anti-STAT3 antibody and loaded for Western blotting. Input samples from equal amounts of proteins blotted for EGFR, STAT3, nucleolin, and α-tubulin are shown as loading and fractionation controls. N: nuclear fraction, C: cytosolic fraction, IB: immunoblot.

To confirm the interaction of EGFR with STAT3 in the nucleus under the regulation of LMP1 at the cellular sublocalization level, co-IP and Western blotting were performed from both cytosolic and nuclear fractions. Cytosolic fractions and nuclear extracts were prepared from CNE1 and CNE1-LMP1 cells, and a co-IP was performed with anti-EGFR (Figure 2A) or anti-STAT3 (Figure 2B) specific antibodies. Nucleolin was used as a control for nuclear extractions while α-tubulin was regarded as a cytosolic extraction control (input panels of Figure 2A). Immunoprecipitation with anti-EGFR antibody in Figure 2A shows that EGFR interacted with STAT3 in both the cytoplasm and nucleus, while LMP1 increased the presence of an EGFR and STAT3 immunocomplex in the nucleus. The IgG control did not detect an EGFR and STAT3 immunocomplex. Using an anti STAT3 antibody, Figure 2B further confirmed that STAT3 interacted with EGFR and that LMP1 promoted the interaction of EGFR with STAT3 in the nucleus. Taken together, these data indicate that LMP1 increased the accumulation of EGFR and STAT3 in the nucleus and shifted the interaction of EGFR with STAT3 from the cytosolic fraction into the nucleus of NPC cells.

### LMP1 activated the activity of cyclin D1 promoter by the EGFR and STAT3 pathways

**Figure 3:**
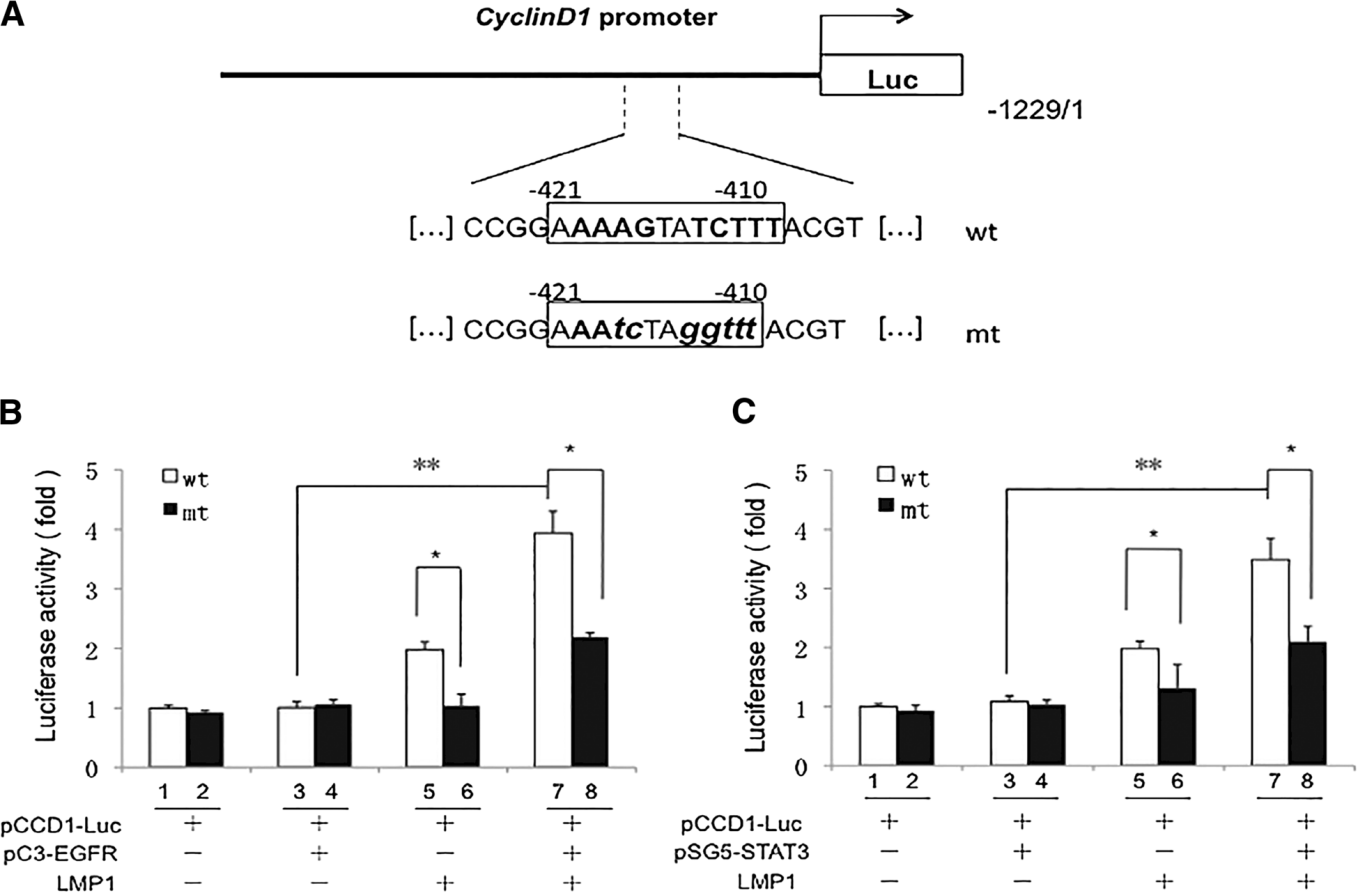
**Identification of an EGFR and STAT3 response element in the cyclin D1 promoter. (A)** Schematic diagram of mutant cyclin D1 promoter constructs are shown. The expansion for EGFR and STAT3 binding site illustrates the wild-type sequence and frames the nucleotides replaced by mutations. **(B-C)** Dual luciferase-reporter assays were performed in LMP1-negative and LMP-positive CNE1 cells after co-transfection of a wild type or mutant cyclin D1 promoter-reporter construct, plasmids expressing wild-type EGFR or STAT3, and a Renilla luciferase transfection control plasmid. The fold induction by EGFR and STAT3 is displayed as the ratio of promoter activity obtained with wild-type compared to the DNA-binding mutant. (mean ± SD, *n* = 3, *p < 0.05, **p < 0.01). mt: mutation, wt: wild-type.

Because cyclin D1 contains both EGFR and STAT3 binding sites adjacent within three nucleotides [31], we addressed whether nuclear accumulation and the interaction between EGFR and STAT3 at the cyclin D1 promoter was under the regulation of the oncoprotein LMP1. The effect of LMP1 on the transcriptional activation of cyclin D1 was examined using a luciferase reporter construct, pCCD1-wt-Luc, driven by the cyclin D1 promoter that contained both EGFR and STAT3 binding sites (Figure 3A). First, we constructed a mutant cyclin D1 promoter luciferase reporter plasmid, pCCD1-mt-Luc, to which no transcription factors would bind at a cyclin D1 promoter region according to a database search (TFSEARCH, http://www.cbrc.jp/research/db/TFSEARCH) (Figure 3A). Then, we transfected the plasmid into CNE1 and CNE1-LMP1 cells, and LMP1 increased the cyclin D1 promoter activity while the mutant cyclin D1 promoter decreased the cyclin D1 promoter activity (column 5 and column 6 of Figure 3B). As shown in Figure 3B, EGFR increased the luciferase expression in CNE1-LMP1 cells (column 7) but not in CNE1 cells (column 3). Mutations in the cyclin D1 promoter greatly (column 6) were attenuated its transcriptional activity in the presence of LMP1 while EGFR rescued the cyclin D1 promoter activity partially (column 8), indicating that LMP1 positively regulates the activity of the cyclin D1 promoter under EGFR. Furthermore, data in Figure 3C demonstrate that STAT3 increased the activity of the cyclin D1 promoter in the presence of LMP1 (column 7 of Figure 3C) while the cyclin D1 promoter activity were decreased greatly after mutating the EGFR and STAT3 binding sites in the Cyclin D1 promoter (column 8 of Figure 3C), further indicating that LMP1 upregulates the activity of the cyclin D1 promoter through STAT3.

### Inhibitors of both EGFR and STAT3 signaling pathways attenuated LMP1-augmented cyclin D1 promoter activities and protein levels

**Figure 4:**
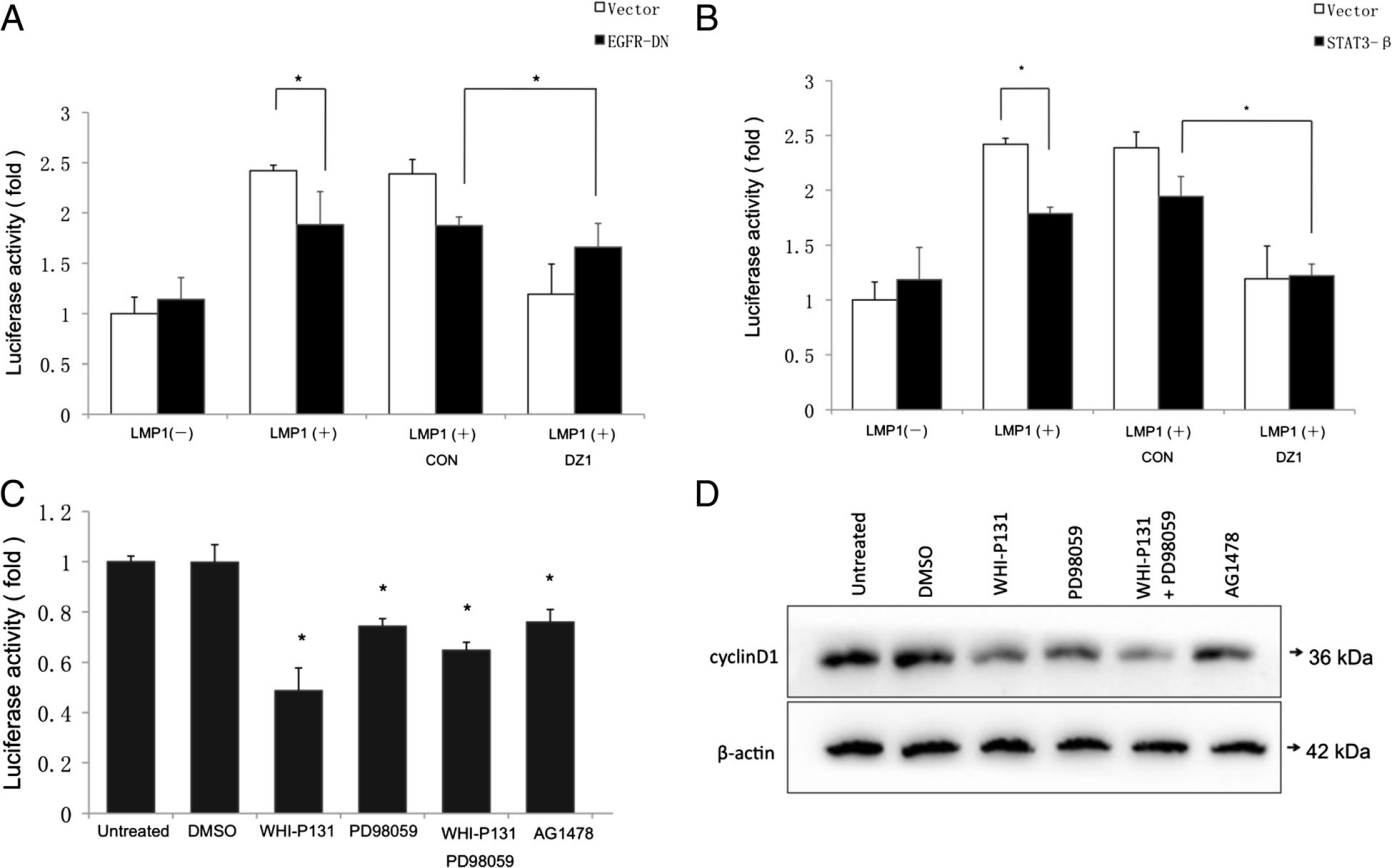
**Inhibitors and dominant negative mutants targeting the EGFR and STAT3 pathways attenuated LMP1-augmented cyclin D1 promoter activity. (A-B)** Stable expression of EGFR-DN and STAT3β inhibited the LMP1-increased activity of cyclin D1. The indicated NPC cell lines were transfected with a cyclin D1 promoter-reporter construct, a Renilla luciferase transfection control plasmid, and an EGFR-DN or STAT3-β expression plasmid. Twenty-four hrs. after transfection, the cells were treated with DNAzymes or a control oligo (2 μM) for 12 hrs. Cells were harvested at 36 hrs. after transfection and subjected to the luciferase assay. Firefly luciferase was measured and normalized to Renilla luciferase activity. The results were expressed as fold induction of the reporter activity in vector-transfected CNE1 cells, which was assigned a value of 1. (mean ± SD, *n* =3, *p < 0.05) **(C)** WHI-P131, PD98059 and AG1478 inhibited the activity of cyclin D1 induced by stable expression of LMP1. CNE1-LMP1 cells were transfected with a cyclin D1 promoter-reporter construct and a Renilla luciferase plasmid as an internal control. Twenty-four hrs. after transfection, the cells were treated with WHI-P131, PD98059, AG1478 or 0.1% DMSO for 2 hrs. The cells were harvested at 26 hrs. after transfection and subjected to the luciferase assay. An empty firefly reporter vector served as a control (*n* = 3). * *p* < 0.05. **(D)** WHI-P131, PD98059 and AG1478 inhibited the expression of cyclin D1 induced by stable expression of LMP1. The cells were harvested for Western Blot at 8 hrs. after the treatment of WHI-P131, PD98059, AG1478 or 0.1% DMSO. β-actin was served as an internal control.

Abnormal cell cycle regulation due to Cyclin D1 overexpression is a common occurrence in human cancers (including NPC), and both EGFR and STAT3 could target cyclin D1 promoter activity [31,35,46]. To further confirm whether the EGFR signaling pathway affects the activity of the cyclin D1 promoter directly, a dominant-negative (DN) variant of EGFR lacking 533 amino acids of the cytoplasmic domain, EGFR-DN [47], was used. The mutant is able to block signaling stemming from several members of the ErbB family and other receptor tyrosine kinases (RTKs). Meanwhile, a specific DNAzyme DZ1 that is targeted to the transmembrane domains of LMP1 [19] decreased the level of LMP1 expression. Figure 4A demonstrated that both DZ1 and EGFR-DN decreased the activity of the cyclin D1 promoter in the presence of LMP1. However, in the presence of EGFR-DN, DZ1 had almost no inhibitory effect on the cyclin D1 promoter activity. STAT3β lacks 55-residues in the C-terminal transactivation domain that is present in STAT3α. Instead, seven unique C-terminal residues act as their full-length counterpart by virtue of missing the C-terminal transactivation domain [44]. Additionally, Figure 4B shows that STAT3β attenuated cyclin D1 promoter activity. In contrast DZ1 inhibitory effect was intact in the presence of STAT3β. Nevertheless DZ1 and STAT3β inhibitory effects are not synergistic.

Nuclear accumulation of EGFR and STAT3 is dependent on the activation of the related signaling pathways. CNE1-LMP1 cells were treated with the small molecule inhibitor WHI-P131, a specific inhibitor of STAT3 phosphorylation at residue tyrosine 705 and serine 727. Both the promoter activity (Figure 4C) and the protein level (Figure 4D) of cyclin D1 decreased greatly upon WHI-P131 treatment. Treatment with PD98059, a chemical inhibitor that blocks the nuclear translocation of STAT3, also decreased cyclin D1 promoter activity (Figure 4C) and protein expression (Figure 4D). On the other hand, the data in Figure 4C and Figure 4D indicated that AG1478, an EGFR specific tyrosine kinase inhibitor, decreased the transcriptional activity of the cyclin D1 promoter and protein level. WHI-P131 was less efficient in the presence of PD98059 in cyclin D1 transcription (Figure 4C) but not cyclin D1 protein level (Figure 4D). siSTAT3 or WHI-P131 induced a stronger inhibition of cyclin D1 promoter activity than siEGFR or AG1478. Taken together, these data suggest that both EGFR and STAT3 signaling pathways are involved in the transcriptional activity of Cyclin D1 promoter and protein levels.

### LMP1 regulated the nuclear EGFR and STAT3 binding to the cyclin D1 promoter region directly

**Figure 5:**
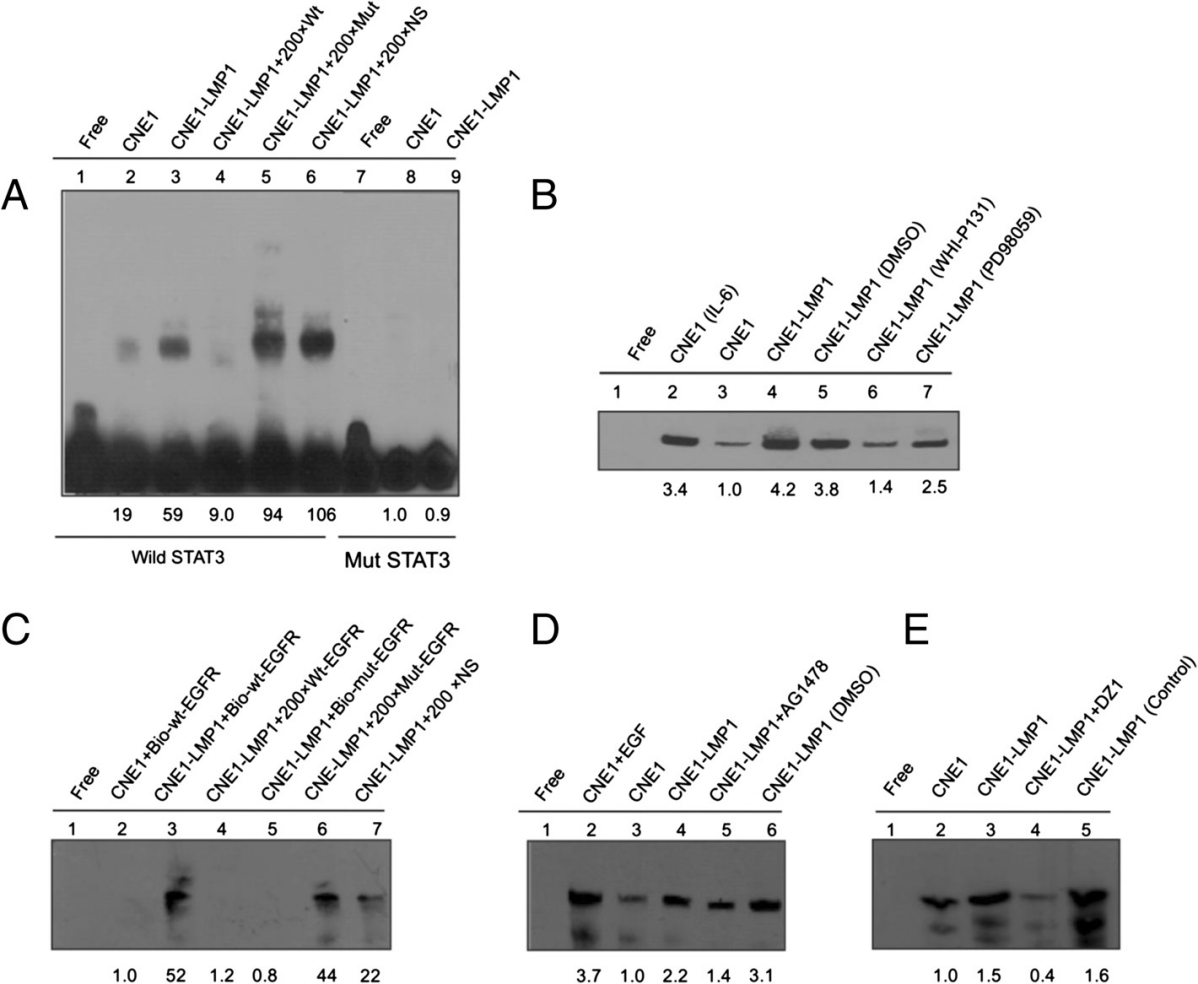
**LMP1 increased the binding ability of transcription factors EGFR and STAT3 to cyclin D1 promoter *in vitro*. (A)** STAT3 binding activities within the cyclin D1 promoter were examined by EMSA. A biotin-labeled wild-type STAT3 oligonucleotide probe was incubated with nuclear extracts of CNE1 and CNE1-LMP1 cells in the presence of a 200-fold excess of unlabeled wild-type STAT3 (lane 4), unlabeled mutant STAT3 oligonucleotides (lane 5), or noncompetitive unlabeled NF-κB oligonucleotide (NS, lane 6). Biotin-labeled mutant STAT3 oligonucleotide probe was incubated with nuclear extracts of the indicated NPC cell lines (lanes 8–9). **(B)** Ten micrograms of nuclear extracts were pre-incubated with biotin-labeled STAT3 oligonucleotide probe in the presence of inhibitors directed against different phosphorylation sites of STAT3 (indicated above each lane). **(C)** The biotin-labeled wild-type EGFR oligonucleotide probe was incubated with nuclear extracts of CNE1 and CNE1-LMP1 cells in the presence of a 200-fold excess of unlabeled wild-type EGFR (lane 4), unlabeled mutant EGFR oligonucleotides (lane 6) or noncompetitive unlabeled NFκB oligonucleotide (NS, lane 7), and then EGFR DNA binding activities were examined by EMSA. **(D-E)** The nuclear extracts of CNE1 and CNE1-LMP1 cells were pre-incubated with biotin-labeled EGFR oligonucleotide probe in the presence of inhibitors AG1478, directed against phosphorylation of EGFR, or DNAzyme 1 (DZ1), targeting LMP1. RD: relative density.

Next, we addressed whether the nuclear interaction of EGFR and STAT3 associates with the cyclin D1 promoter directly using electrophoresis mobility shift assay (EMSA) in CNE1 and CNE1-LMP1 cells. The probes, which contain EGFR or STAT3 binding sites according to the previous report [31], were labeled with biotin. As shown in Figure 5A, we found significant binding of nuclear protein to cyclin D1 (lane 2) while LMP1 promoted more nuclear protein binding (lane 3), indicating that LMP1 promoted STAT3 binding to the cyclin D1 promoter. The complex in CNE1-LMP1 cells was abolished by adding cold STAT3 binding sequence (Figure 5A, lane 4) but not by a mutation in the STAT3 binding sequence (Figure 5A, lane 5) or a nonspecific binding sequence (Figure 5A, lane 6). After we mutated the plasmid containing functional mutated cyclin D1 promoters, we could not detect the band in either CNE1 or CNE1-LMP1 cells (lanes 8 and 9 of Figure 5A). After the CNE1 cells were treated with IL-6 to induce STAT3 activation, we observed STAT3 binding in the cyclin D1 promoter (Figure 5B). After the CNE1-LMP1 cells were treated with the STAT3 inhibitors WHI-P131 and PD98059 (Figure 5B), we observed that STAT3 binding in the cyclin D1 promoter decreased. Taken together, LMP1 promoted STAT3 binding to the Cyclin D1 promoter.

To address whether nuclear EGFR is involved with the cyclin D1 promoter directly, we mutated the cyclin D1 promoter sequence such that no transcription factor binds. As shown in Figure 5C, biotin-labeled wild-type EGFR oligonucleotide and nuclear EGFR formed a specific complex in CNE1- LMP1 cells (Figure 5C lane 3). With a mutated EGFR probe, no specific complex band was present (Figure 5C lane 5), whereas a weak band was detected in CNE1 cells. Formation of this complex from CNE1- LMP1 cells was blocked by competition with the cold EGFR (Figure 5C lane 4) but not by the mutated EGFR or nonspecific nucleotide NF-κB (Figure 5C lanes 6 and 7). After blocking the EGFR signaling pathway with the small molecule inhibitor AG1478, the band indicating a complex was weaker in the CNE1-LMP1 nuclear proteins (Figure 5D). To confirm that LMP1 controlled the cyclin D1 promoter, the CNE1-LMP1 cells were treated with DZ1, which is a specific LMP1-targeted DNAzyme construct [19]. Data in Figure 5E showed that the complex band of biotin-labeled EGFR nucleotide with nuclear protein weakened in CNE1-LMP1 cells after treatment with DZ1. Taken together, these results show that LMP1 regulates the binding capacity of EGFR, STAT3 to the cyclin D1 promoter region *in vitro*.

### LMP1 induced EGFR and STAT3 to activate cyclin D1 gene expression

**Figure 6:**
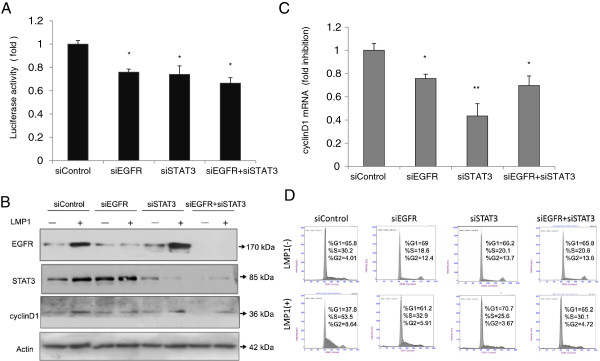
**Cyclin D1 expression is reduced in CNE1-LMP1 cells after treatment with EGFR siRNA and STAT3 siRNA. (A)** Dual luciferase-reporter assays were performed in CNE1-LMP1 cells after co-transfection with either control siRNA (siControl), EGFR siRNA (siEGFR), or STAT3 siRNA (siSTAT3) in addition to cyclin D1 promoter-reporter constructs and a Renilla luciferase transfection control plasmid. Firefly luciferase was measured and normalized to Renilla luciferase activity. The fold change in cyclin D1 expression by the indicated siRNA is displayed in each case. The control siRNA served as a non-targeting control. (mean ± SD, *n* =3, *p < 0.05) **(B)** The cells were incubated with medium containing the indicated siRNAs for 72 h. Total RNA was isolated from the cells and subjected to real-time PCR, using specific primers designed to amplify cyclin D1. β-actin mRNA served as an internal control. (mean ± SD, *n* =3, *p < 0.05, **p < 0.01). **(C)** Western Blot was performed in CNE1-LMP1 cells after co-transfection with the indicated siRNAs for 72 h. β-actin was served as an internal control. **(D)** FACS was performed in CNE1 and CNE1-LMP1 cells after co-transfection with the indicated siRNAs for 72 h. The data are presented from three independent experiments.

To address whether EGFR and STAT3 may be involved in cyclin D1 activity, we knocked down EGFR or STAT3 with siRNA. After we introduced EGFR siRNA or and STAT3 siRNA in CNE1-LMP1 cells (Figure 6A), the cyclin D1 promoter activity decreased compared to treatment with nonspecific siRNA (siControl). We also used siRNA to further confirm the roles of EGFR and STAT3 in the regulation of cyclin D1 mRNA. Knockdown of EGFR and STAT3 with siRNA decreased the cyclin D1 mRNA level in CNE1-LMP1 cells (Figure 6B). We could not detect a stronger effect of the combined knockdown of EGFR and STAT3 on cyclin D1 promoter activity or mRNA level. To further confirm that both EGFR and STAT3 may be involved in the cyclin D1 protein, we detected the cyclin D1 protein level after we knocked down EGFR or STAT3 with siRNA. Data in Figure 6C showed that knockdown of EGFR and STAT3 with siRNA decreased the cyclin D1 protein level in CNE1-LMP1 cells. To further address how EGFR or STAT3 affects the cell cycle, we performed FACS analysis on the CNE1-LMP1 cells after knockdown of EGFR, STAT3 or both. Data in Figure 6D indicated that the depletion of EGFR, STAT3 or both proteins altered the cell cycle distribution especially at S phase with the stimulation of LMP1. Taken together, these findings demonstrate that both EGFR and STAT3 are essential for cyclin D1 expression in the presence of LMP1.

## Discussion

cyclin D1 over-expression is important in the development and progression of numerous cancers [48]. Regulation of the cyclin D1 protein level is one of the critical aspects in cell proliferation and tumor development [49], indicating that cyclin D1 may be regarded as a therapeutic target in cancer [50]. Cyclin D1 is upregulated expression in NPC [51]. Overexpressed cyclin D1 in NPC increases the risk of tumor formation and local disease recurrence [52]. Although cyclin D1 is known to be a target gene of EGFR and STAT3 [46,53-56], its transcriptional regulation remains elusive after the infection of virus. Our previous study reported that LMP1 encoded by EBV could regulate the nuclear accumulation of EGFR and that nuclear EGFR could bind to the promoters of cyclin D1 and cyclin E to accelerate the G1/S phase transition. Another report showed that EBV LMP1 signals through the Janus kinase 3 (JAK3) and ERK1/2 pathways upon the activation of STAT3 and STAT transactivation to induce expression of VEGF [34]. Overexpressed EGFR could activate specifically and persistently STAT3 after the decrease TGF-beta signaling pathway [57]. The key contribution of the present study is to provide a link between signaling via LMP1/EGFR and LMP1/STAT3, which is consistent with the previous findings that EBV LMP1 could promote the expression of EGFR [58,59].

The mechanism by which EBV LMP1 induces EGFR and STAT3 to enhance the promoter activity and expression of cyclin D1 involves physical and functional interaction between EGFR and STAT3. This observation is in agreement with other reports that nuclear EGFR interacts with transcription factors, such as STAT3, E2F1, STAT5 and TIF2 to induce the expression of some target genes in various cancers [31,40,60-63]. Nuclear EGFR-targeted genes including cyclin D1 [54,64], iNOS, B-Myb, Aurora A and COX-2, have been reported, yet these studies did not support cyclin D1 as the target gene co-regulated by EGFR and other transcription factors after the infection of EBV, such as in the work of EGFR and STAT3 co-affecting on iNOS and STAT1 in breast cancer [31,57]. Together, these findings suggest the EGFR-STAT3 axis signaling pathway is critical in regulating cellular transcriptional and biologic properties in different carcinomas in response to diverse carcinogens such as virus infection.

Our previous studies reported EBV LMP1 induces in both expression and phosphorylation of EGFR in a dose-dependent manner [21,45], and other authors demonstrated EGFR that accumulated in the nucleus of breast carcinoma cell lines and esophageal cancer tissues was highly tyrosine-phosphorylated [54,65]. Meanwhile, we found EBV LMP1 expressing cells exhibited more nuclear accumulation of Tyr 705-phophorylated STAT3 (pY-STAT3) [35,45]. EGFR physically interacts and functionally cooperates with STAT3 at both the cytoplasmic and nuclear levels. As reported, EGFR and phosphorylated STAT3 were strongly expressed in the nucleus of cancer cells in surgical and biopsy specimens of nasopharyngeal tissues from NPC patients in southern China [35,66], suggesting that EGFR- and STAT3-dependent mechanisms are important for carcinogenesis.

It has been shown that LMP1 induces cyclin D1 expression through EGFR in NPC cells [23]. The present study show that the promoter activity and mRNA expression level of cyclin D1 in LMP1-expressing cells could be decreased by co-transfecting the plasmids of mutated EGFR/STAT3 or siRNA for EGFR and siSTAT3. However, we did not find the cooperative effect of siEGFR and siSTAT3 at both mRNA and protein levels of cyclin D1. We provide the evidence showing cyclin D1 might be modulated by STAT3 induced by EBV LMP1, illustrating the importance of the JAK/STAT signaling pathway on EBV LMP1 induced cyclin D1 transcription and expression.

The current standard therapy for NPC is radical radiotherapy for early stage disease and concurrent chemoradiotherapy for advanced disease [67,68]. EGFR and STAT3 are good targets for cancers treatment. Thus, agents such as the anti-EGFR antibody cetuximab, the EGFR tyrosine kinase inhibitor gefitinib, and STAT3 inhibitors (such as S3I-201 or JSI-124) could be used in preclinical models or each phase of clinical trials [69-71]. Interestingly, a novel STAT3 inhibitor S3I-1747 selectively interrupt the interaction of EGFR and STAT3 directly [72]. Those reports also suggested that either an anti-EGFR or anti-STAT3 agent might be a potent chemopreventive agent for patients with anti-invasion and anoikis-sensitizing activities. Therapies such as monoclonal antibodies and tyrosine kinase inhibitors targeting EGFR have demonstrated limited anti-tumor efficacy [71,73]; however, reports of combined targeting of EGFR and STAT3 are few. Recently, EBV LMP1-specific DNAzyme, DZ1, inhibits the majority of oncogenic signaling pathways converging on sets of transcription factors that ultimately control gene expression patterns resulting in tumor formation, progression, and metastasis. [19] Our data showed that DZ1 can inhibit EBV LMP1-induced promoter activity of cyclin D1 via EGFR or STAT3 and that DZ1 enhanced cyclin D1 promoter inhibition based on experiments with mutants of EGFR or STAT3. These results suggest that combining inhibitors for EGFR/STAT3 and DZ1 in LMP-expressing cancers may be a promising therapeutic strategy. The combination of Src and EGFR inhibition with Gemcitabine treatment in STAT3-mediated therapy-resistant pancreatic tumors was also effective at inhibiting the growth of xenografts of both therapy-sensitive and -resistant pancreatic cancer cells *in vivo* without increasing toxicity [73]. It is possible that EGFR and STAT3, individually or as a pair, contribute to tumor progression. Alternatively, crosstalk between signaling pathways provides a potential route to overcome the blockade of a single or double targeted therapies, but this can be overcome by the blockade of multiple targets. Our data provide further evidence that the combination of three inhibitors may be efficacious for cancer, and more extensive investigation will be required.

In summary, we found that EBV LMP1 enhances the transcriptional activity and mRNA level of the cyclin D1 gene in CNE1 cells. This underlying mechanism for cyclin D1 regulation involves regulated binding of EGFR and STAT3 in the cyclin D1 promoter region as well as increasing the promoter activity of the cyclin D1 gene. Such a mechanism may partially contribute to the proliferation and growth of tumor cells with an LMP1-induced increase in the nuclear accumulation of EGFR and STAT3.

## Competing interests

The authors declare that they have no competing of interests.

## Authors’ contributions

Conceived and designed the experiments: YT YC. Performed the experiments: YX, SY, QY, XL, BY and LC. Analyzed the data: YX, SY, QY, XL, BY and LC. Contributed reagents/materials/analysis tools: SY and LC. Wrote the paper: YX, YT and YC. All authors read and approved the final manuscript.

## Supplementary Material

Additional file 1: Figure S1 LMP1 promoted the interaction of phosphorylated EGFR and phosphorylated STAT3. Two mg of protein from cell lysates were immunoprecipitated with an anti-phosphorylated EGFR antibody (p-EGFR) and analyzed by Western blotting with a phosphorylated STAT3 (p-STAT3) and p-EGFR antibodies. Negative controls included immunoprecipitation with an unrelated antibody (IgG). Click here for file
