# Supplementary material for: Epstein-Barr Virus encoded LMP1 regulates cyclin D1 promoter activity by nuclear EGFR and STAT3 in CNE1 cells
Source: J Exp Clin Cancer Res. 2013 Nov 13;32(1):90. doi: 10.1186/1756-9966-32-90 (PMC3843577; doi:10.1186/1756-9966-32-90)
Supplement: Additional file 1: Figure S1 — LMP1 promoted the interaction of phosphorylated EGFR and phosphorylated STAT3. Two mg of protein from cell lysates were immunoprecipitated with an anti-phosphorylated EGFR antibody (p-EGFR) and analyzed by Western blotting with a phosphorylated STAT3 (p-STAT3) and p-EGFR antibodies. Negative controls included immunoprecipitation with an unrelated antibody (IgG). [file 1756-9966-32-90-S1.ppt]

## Slide 1
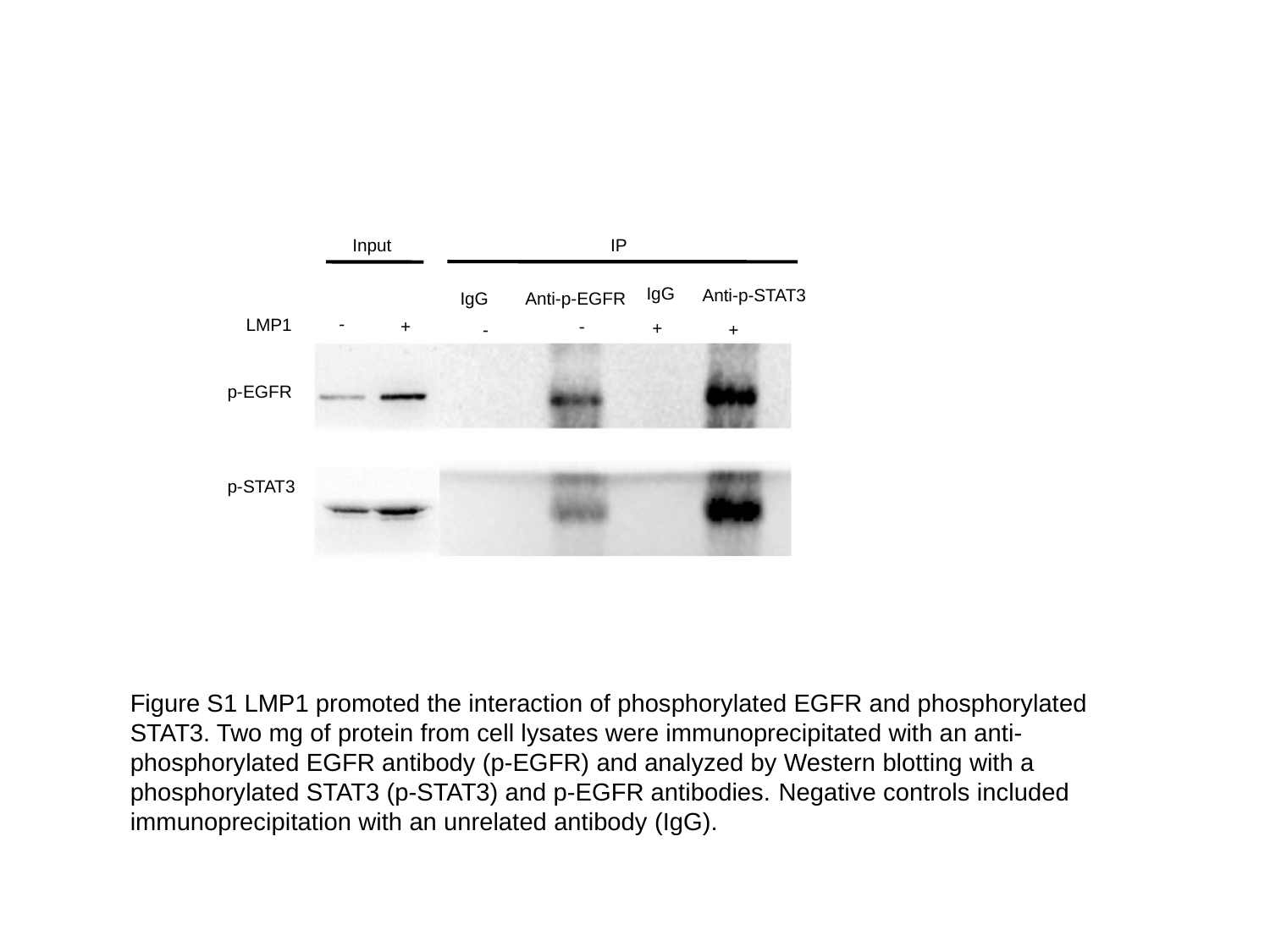

Input
IP
IgG
Anti-p-STAT3
IgG
Anti-p-EGFR
-
LMP1
+
-
+
-
+
p-EGFR
p-STAT3
Figure S1 LMP1 promoted the interaction of phosphorylated EGFR and phosphorylated STAT3. Two mg of protein from cell lysates were immunoprecipitated with an anti-phosphorylated EGFR antibody (p-EGFR) and analyzed by Western blotting with a phosphorylated STAT3 (p-STAT3) and p-EGFR antibodies. Negative controls included immunoprecipitation with an unrelated antibody (IgG).

## Slide 2
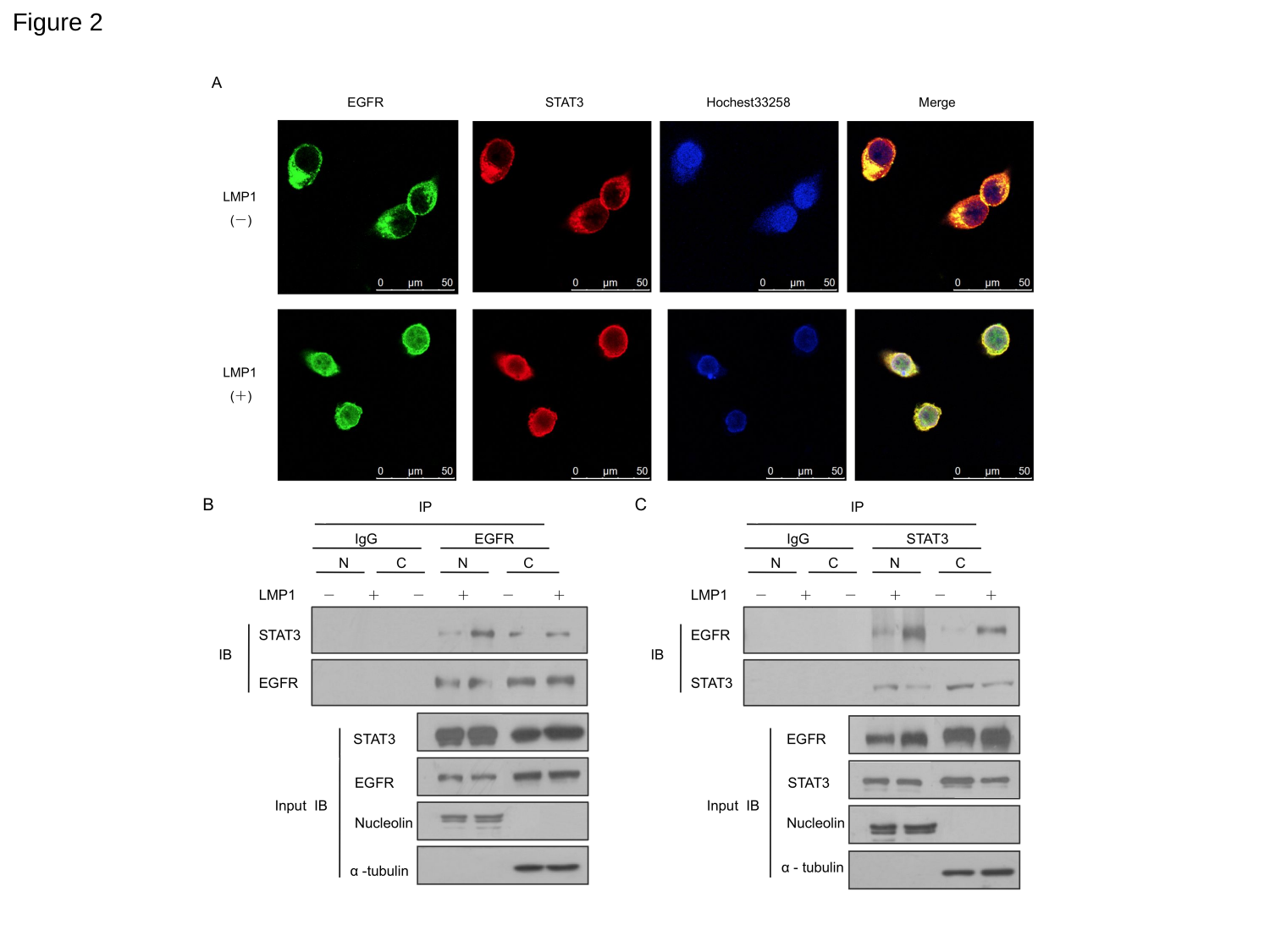

Figure 2

## Slide 3
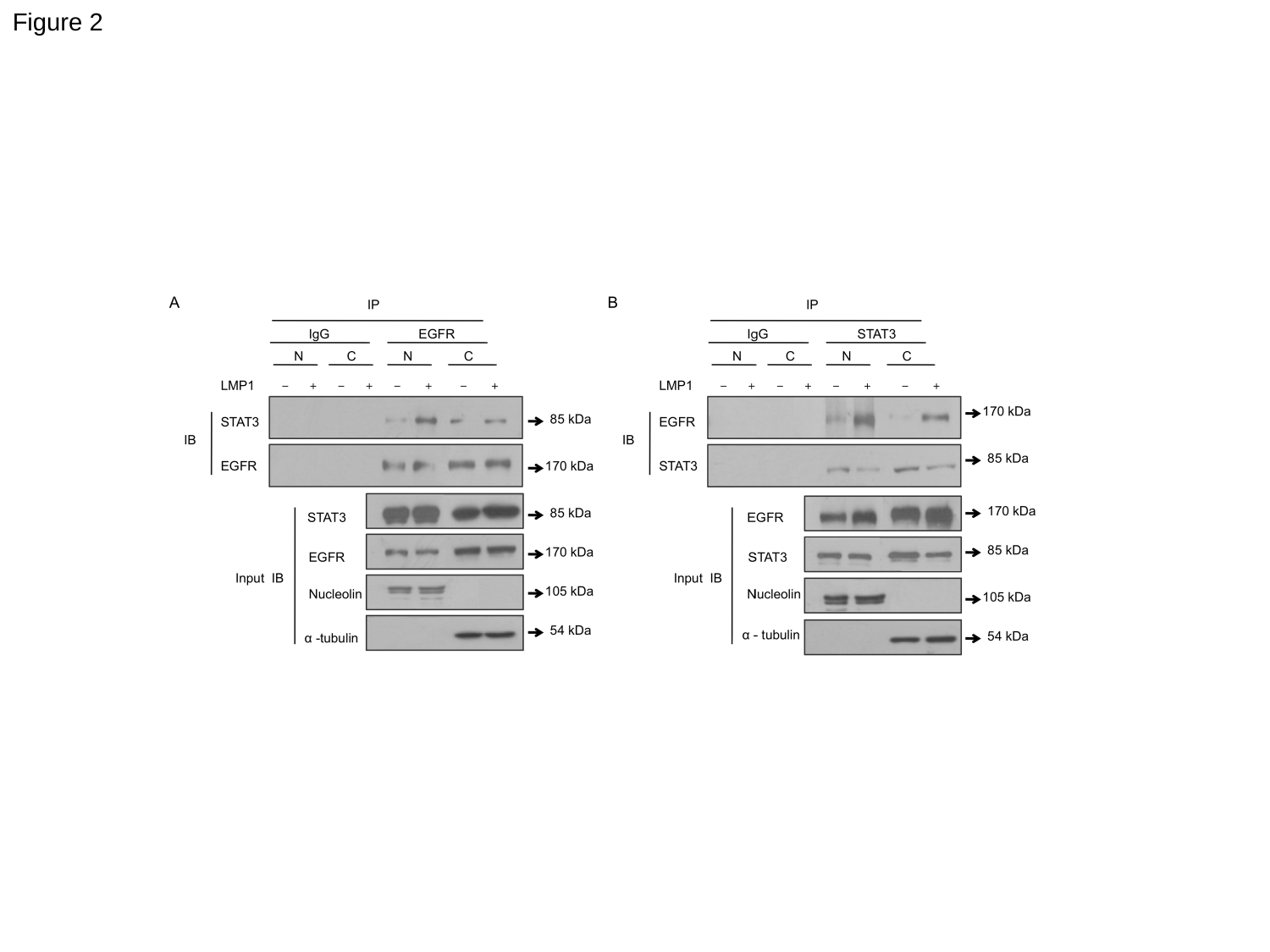

Figure 2
